# CD40 stimulation via CD40 ligand enhances adenovirus‐mediated tumour immunogenicity including ‘find‐me’, ‘eat‐me’, and ‘kill‐me’ signalling

**DOI:** 10.1111/jcmm.18162

**Published:** 2024-03-17

**Authors:** Sedigheh Naseri, Mariela Mejia Cordova, Jessica Wenthe, Tanja Lövgren, Emma Eriksson, Angelica Loskog, Gustav J. Ullenhag

**Affiliations:** ^1^ Department of Immunology, Genetics and Pathology (IGP), Science for Life Laboratories Uppsala University Uppsala Sweden; ^2^ Lokon Pharma AB Uppsala Sweden; ^3^ Department of Oncology Uppsala University Hospital Uppsala Sweden

## Abstract

Immunostimulatory gene therapy using oncolytic viruses is currently evaluated as a promising therapy for cancer aiming to induce anti‐tumour immunity. Here, we investigate the capacity of oncolytic adenoviruses (LOAd) and their transgenes to induce immunogenicity in the infected tumour cells. Oncolysis and death‐related markers were assessed after infection of eight human solid cancer cell lines with different LOAd viruses expressing a trimerized, membrane‐bound (TMZ)‐CD40L, TMZ‐CD40L and 41BBL, or no transgenes. The viruses induced transgene expression post infection before they were killed by oncolysis. Death receptors TRAIL‐R1, TRAIL‐R2 and Fas as well as immunogenic cell death marker calreticulin were upregulated in cell lines post infection. Similarly, caspase 3/7 activity was increased in most cell lines. Interestingly, in CD40^+^ cell lines there was a significant effect of the TMZ‐CD40L‐encoding viruses indicating activation of the CD40‐mediated apoptosis pathway. Further, these cell lines showed a significant increase of calreticulin, and TRAIL receptor 1 and 2 post infection. However, LOAd viruses induced PD‐L1 upregulation which may hamper anti‐tumour immune responses. In conclusion, LOAd infection increased the immunogenicity of infected tumour cells and this was potentiated by CD40 stimulation. Due to the simultaneous PD‐L1 increase, LOAd viruses may benefit from combination with antibodies blocking PD1/PD‐L1.

## 1 INTRODUCTION

Oncolytic adenoviruses utilize multiple mechanisms‐of‐action to eliminate tumour cells. Replication of the viral genome and the generation of new particles causes tumour cell death by oncolysis, which leads to the release of viral particles that can initiate subsequent replication in neighbouring cells.^1^ Oncolysis is characterized by immunogenic cell death where damage‐associated molecule patterns (DAMPs) are exposed on the cell surface or released from the dying cells with tumour antigens.^2^, ^3^, ^4^ DAMPs attract and induce activation of immune cells, including dendritic cells (DCs), which is a critical step initiating an anti‐viral or anti‐tumour response and are also referred to as find‐me and eat‐me signals.^5^ The immune activation can be potentiated by arming the virus with immunostimulatory transgenes.^6^, ^7^, ^8^

Lokon Oncolytic Adenovirus (LOAd) is a platform of serotype 5/35 chimera viruses that are armed with tumour microenvironment‐modulating transgenes such as the immune stimulators trimerized, membrane‐bound CD40 ligand (TMZ‐CD40L) and 4‐1BB ligand (4‐1BBL).^9^, ^10^, ^11^ LOAd viruses utilize CD46 as viral entry receptor. Due to a deletion in the E1A region of the viral genome, replication is restricted to cells with a dysregulated retinoblastoma pathway. Further, it has a deletion of E3 regions to reduce the capacity of the virus to protect the infected cells from T cell‐mediated killing.^10^, ^12^ LOAd703 (delolimogene mupadenorepvec) is the first virus from the LOAd platform to reach clinical investigation (NCT02705196, NCT03225989, NCT04123470 and NCT03555149). LOAd703 is armed with both TMZ‐CD40L and 4‐1BBL. CD40L has a central role in the activation of the immune system and is critical for B cells and DCs and indirectly for T cell priming via the matured DCs.^13^ Furthermore, CD40L signalling in CD40 positive tumour cells can lead to induction of apoptosis.^14^ 4‐1BBL induces activation and proliferation of T cells and NK cells and is involved in the generation of T cell memory.^15^, ^16^ We have previously shown the immunostimulatory effect of LOAd703 in vitro and in an in vivo model of malignant melanoma, were LOAd703 augmented immune checkpoint blockade, in particular, anti‐TIM3 and anti‐PD‐L1.^9^, ^17^, ^18^

The oncolytic capacity of LOAd viruses has previously been evaluated in human cell lines derived from pancreatic cancer, multiple myeloma and B cell lymphoma.^19^, ^20^ However, the contribution of the LOAd viruses, with and without different transgenes, to the tumour cell immunogenicity has so far not been investigated. The aim of this study was to elucidate the killing capacity and immunogenicity induced in the tumour cell by the virus itself and by the transgenes TMZ‐CD40L and 4‐1BBL.

## 2 MATERIALS AND METHODS

### 2.1 Cell lines

T24 (bladder carcinoma), SKOV3 (ovarian adenocarcinoma), A549 (lung carcinoma), NCI‐HT29 (colorectal adenocarcinoma) and NCI‐H727 (lung carcinoid) were all purchased from ATCC (Manassas, VA). Mel526 (melanoma cell line) was a kind gift from Dr Magnus Essand and PEA2 (ovarian adenocarcinoma) was a kind gift of Dr Yumeng Mao, both from Uppsala University, Sweden. MiaPaca2 (pancreatic cell line) was a kind gift from Dr Rainer Heuchel (Karolinska Institute, Sweden). T24 and MiaPaCa2 were cultured in Dulbecco's Modified Eagle Medium (DMEM), NCI‐H727, A549, PEA2 and Mel526 were cultured in Roswell Park Memorial Institute (RPMI)‐1640 medium and NCI‐HT29 and Skov3, were cultured in McCoy's 5A medium. All culture media were supplemented with 10% fetal bovine serum (FBS) and penicillin (100 U/mL)/streptomycin (100 μg/mL) (PeSt). For A549 and PEA2, 1% of sodium pyruvate was added to the cultures. All reagents were purchased form Invitrogen, Carlsbad, CA, USA.

### 2.2 Viruses

LOAd viruses are genetically modified adenoviruses of serotype 5 with fibre switched to serotype 35. In addition to the oncolytic capacity, LOAd703 and LOAd700 carry a cassette for immunostimulatory transgenes TMZ‐CD40L (in LOAd703 and LOAd700) and 4‐1BBL (in LOAd703) under a CMV promotor, while LOAd(−) lacks the transgene cassette. The construction of the viruses has previously been described.^9^, ^10^ The viral titers were quantified with a fluorescence‐forming unit (FFU) assay.^21^ LOAd viruses were provided by Lokon Pharma AB, Uppsala, Sweden.

### 2.3 Cell viability assay

Cell lines were infected with 100 FFU/cell of LOAd703, LOAd700, LOAd(−), or left uninfected for 2 h before plating in 96‐well plates at 1 × 10^4^ cells in 200 μL media per well in quadruplicates. After culturing for 48 h at 37°C, 5% CO_2_, 20 μL tetrazolium compound [3‐(4,5‐dimethylthiazol‐2‐yl)‐5‐(3‐carboxymethoxyphenyl)‐2‐(4‐sulfophenyl)‐2H‐tetrazolium, inner salt; MTS] was added (MTS Cell Titre Aqueous One Solution cell proliferation assay kit; Promega, Madison, WI, USA). The plate is incubated for 1 h at 37°C, 5% CO_2_ after which the absorbance was determined at 490 nm with a plate reader using medium control as a blank. The cell viability was calculated comparing the values of infected cells to the uninfected control.

### 2.4 Animal experiments

Animal experiments were approved by local animal ethics committee (Dnr: C54/13 and Dnr 5.8.18‐08932/2019) and performed at Uppsala University. Immunodeficient BalbC nu/nu mice were inoculated with 5–10 million cells/mouse. Animals were treated with intratumoral injections of LOAd703, LOAd(−) or phosphate‐buffered saline (PBS) starting when 50% of the animals had a palpable tumour. Treatments were given twice a week for 3 weeks (six injections in total). Concentration of virus injected was 1 × 10^9^ FFU per animal. The mice were monitored twice a week for tumour growth. The weight was measured once weekly. Mice were sacrificed when the tumour passed an area of 100 mm^2^/1000 mm^3^ or their weight had decreased by 10%.

### 2.5 Phenotype screening

All cell lines were analysed for expression of CD46, CD40, 4‐1BB, the transgenes (CD40L, 4‐1BBL) and other markers at baseline and post LOAd infection. Cells were infected with 10 FFU/cell of LOAd703, LOAd700, LOAd(−), or left uninfected for 48 h before cell supernatants were collected for subsequent analysis and cells were harvested for flow cytometry analysis. Cells were first washed with PBS supplemented with 3 mM EDTA (Thermo Fisher, Waltham, USA) and 0.5% bovine serum albumin (Sigma‐Aldrich, Saint Louis, MO, USA) for blocking of unspecific antibody binding. Cells were stained first with viability dye, Zombie Near IR (APC/−C7) and thereafter with antibodies targeting CD46 (PE, clone TRA‐2‐10), CD40L (BV421, clone 24‐31), CD40 (APC, clone HB14), 4‐1BBL (PE, clone 5F4), 4‐1BB (BV421, clone 4B4‐1), CD95 (FITC, clone DX2), CD261 (PE, clone DJR1), CD262 (APC, clone DJR2‐4 (7‐8)), PDL‐1 (APC, clone 29E.2A3) and Calreticulin (DyLight488, clone FMC75). After incubation, the cells were washed once before being fixed with 1% formaldehyde in PBS containing 3 mM EDTA. Cells were analysed with a BD FACS Canto II (BD Biosciences, San Jose, CA, USA) and the results were evaluated by Flow Jo software (FlowJo LLC, Ashland, OR, USA). All antibodies were purchased from Biolegend (San Diego, CA, USA).

### 2.6 Soluble markers

For detection of soluble markers (sCD40L and ATP) cell supernatants were collected 48 h post infection from cells infected with 10 FFU/cell of different LOAd viruses or from uninfected cells. Soluble CD40L was detected by Human sCD40L Platinum ELISA Kit (eBioscience, San Diego, CA, USA). Briefly, supernatants and known concentrations of provided sCD40L (standard curve) were added to 96 well plates precoated with anti‐CD40L antibodies. After 1 h incubation in room temperature, the plate is washed with wash solution afterwhich detection antibody is added to each well and the plate is incubated for another 1 h in room temperature. After washing the plate, horse radish peroxidase (HRP)‐streptavidin is added to the wells and the incubation is continued for 45 min in room temperature. Finally, enzyme substrate is added and mixed gently for a uniform colour distribution and the plate is incubated for 30 min at room temperature. Stop solution is added to each well and within 60 min the absorbans is evaluated with spectrophotometry at 450 nm. The sample concentration was determined against the standard curve. Extracellular ATP was analysed with ATP Determination Kit (Invitrogen, Waltham, USA). Briefly, cell supernatants and known concentrations of provided ATP (standard curve) were added to 96 well plates. Reaction Buffer was added to the samples and the luminescence evaluated with an luminometer. The background luminescence generated by reaction buffer only was subtracted prior evaluating the samples. The sample concentration was determined against the standard curve.

### 2.7 Caspase 3/7 assay

Cell lines were infected with 50 FFU/cell of LOAd703, LOAd700, LOAd(−) or left uninfected for 2 h before plating in 96 well plates at 1 × 10^4^ cells per well in triplicates. After culturing for 24 h, Caspase 3/7 activity was measured by Caspase‐Glo® 3/7 assay (Promega, Madison, WI, USA). Briefly, Caspase‐Glo 3/7 reagent was added to the 96 well plate with cell samples followed by a 30 min to 3 h long incubation. Active Caspase 3 and 7 can cleave the substrate which liberates free aminoluciferin which is consumed by luciferase to generate signal. Luminescence was recorded with a luminometer.

### 2.8 Statistics

Statistical analysis was performed in GraphPad Prism 9 (La Jolla, CA, USA). One‐way ANOVA with Dunnett's multiple comparison test was used when treatment groups were compared against a control group. Tukey multiple comparison test was used when more than two groups were compared and analysed against each other.

## 3 RESULTS

### 3.1 The virus entry receptor CD46 is expressed in solid malignancies

**FIGURE 1:**
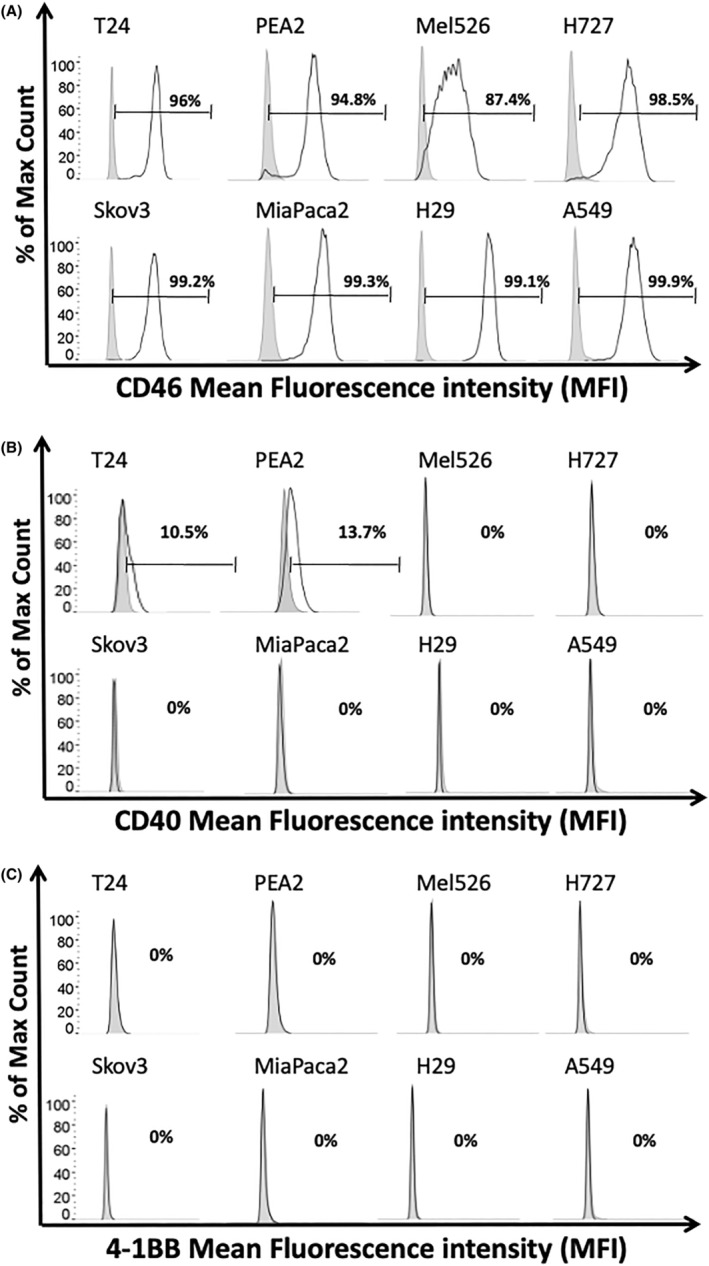
Expression of CD46, CD40 and 4‐1BB in solid cancer cell lines. Human cancer cell lines (T24, PEA2, Mel526, H727, Skov3, MiaPaCa2, H29 and A549) were analysed for expression of CD46 (A), CD40 (B) and 4‐1BB (C) by flow cytometry. Grey filled peaks represent isotype control staining and black lines display CD46, CD40 and 4‐1BB expression. Percent of positive cells are shown for each cell line and marker.

The entry receptor for adenovirus serotype 35 is the complement regulatory receptor CD46.^22^ The LOAd viruses are modified to contain the serotype 35 fibre and therefore only infect CD46 positive cells. In our panel of eight cell lines from various solid malignancies, all expressed high levels of CD46 (Figure 1A). Hence, the cell lines are sensitive to LOAd virus infection. Next, we analysed if the cell lines expressed the receptors for the transgenes expressed by the LOAd viruses. Both the T24 and PEA2 cell lines expressed CD40, the receptor for TMZ‐CD40L, but the others were negative for this receptor (Figure 1B). None of the cell lines expressed 4‐1BB, the receptor for 4‐1BBL (Figure 1C).

### 3.2 Tumours of various origin were susceptible to gene transfer using LOAd viruses

**FIGURE 2:**
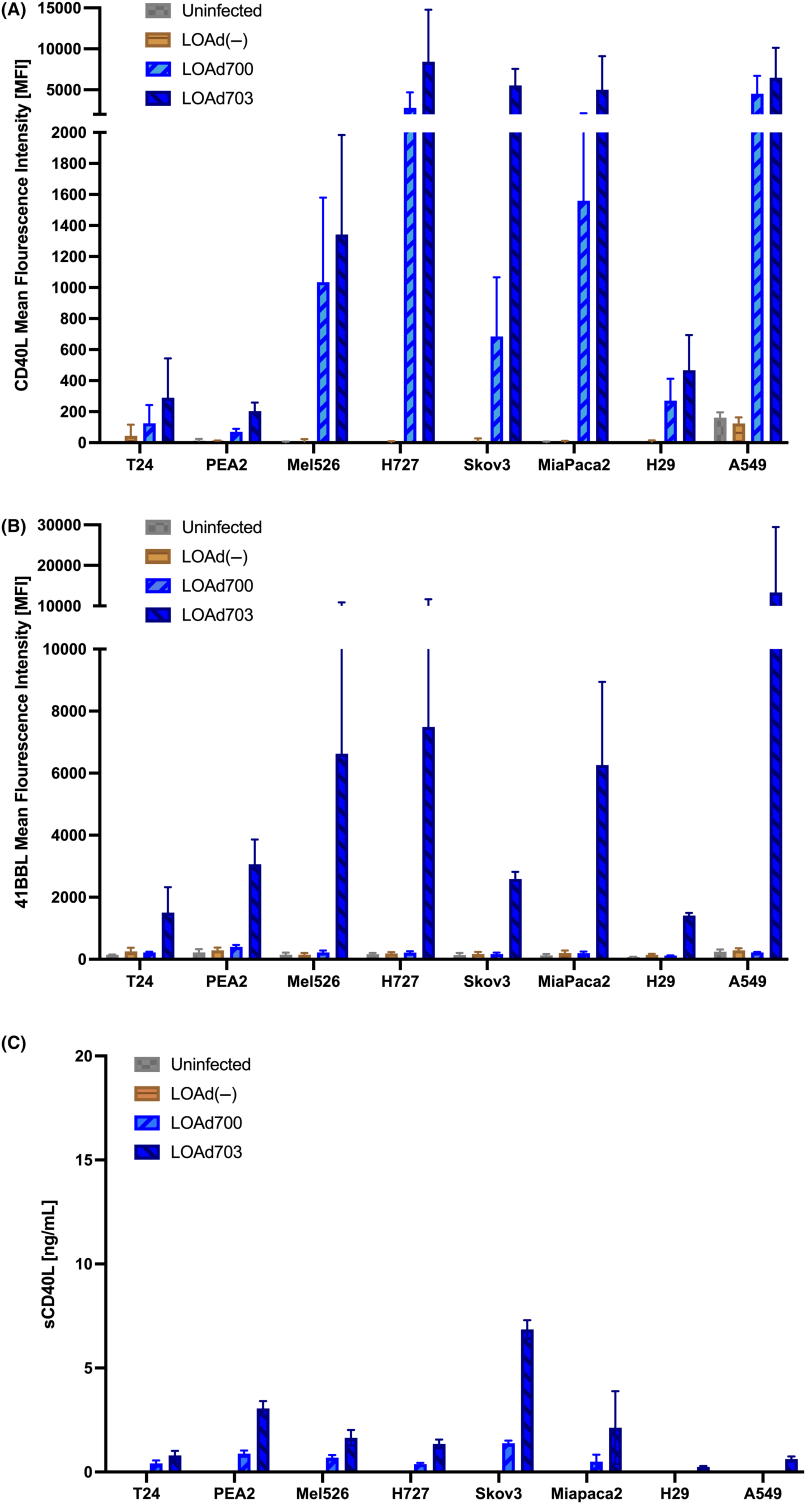
Expression of CD40L and 4‐1BBL post LOAd infection in cancer cell lines. Human cancer cell lines (T24, PEA2, Mel526, H727, Skov3, MiaPaCa2, H29 and A549) were infected with 10 FFU/cell of LOAd(−), LOAd700, LOAd703 or left uninfected. Cells were cultured for 48 h and analysed for expression of CD40L (A) and 4‐1BBL (B) by flow cytometry or for soluble CD40L using ELISA (C). For flow cytometry bar graphs show delta mean fluorescence intensity (MFI) (MFI test‐ MFI isotype) (mean ± SD) of three independent replicates. For ELISA, bars display protein concentration. Statistics were calculated with ordinary one‐way ANOVA with Dunnett's multiple comparisons test.

LOAd viruses efficiently infected all eight cell lines. Both LOAd703‐ and LOAd700‐infected cells expressed TMZ‐CD40L (Figure 2A) while only LOAd703 mediated 4‐1BBL expression (Figure 2B). The cell lines expressed the transgenes at different levels. The lowest expression was found in T24, PEA2 and H29. 4‐1BBL was in general expressed at a higher level in all cells compared to TMZ‐CD40L but that may be due to the construction of the transgene cassette in which 4‐1BBL is placed before TMZ‐CD40L. LOAd(−), which lacks transgenes, did not induce significant expression of CD40L or 4‐1BBL indicating it was not the virus backbone per se that stressed the cells to upregulate these immune receptors. However, a modest increase was noted in T24 that may reflect wild type CD40L. Wild type CD40L can be cleaved and shedded in soluble form. However, sCD40L was low in the infected cells (i.e. on the border of the kit limits) except for LOAd703‐infected SKOV‐3 and PEA2 (Figure 2C).

### 3.3 LOAd‐mediated oncolysis in vitro does not always translate to in vivo tumour control

**FIGURE 3:**
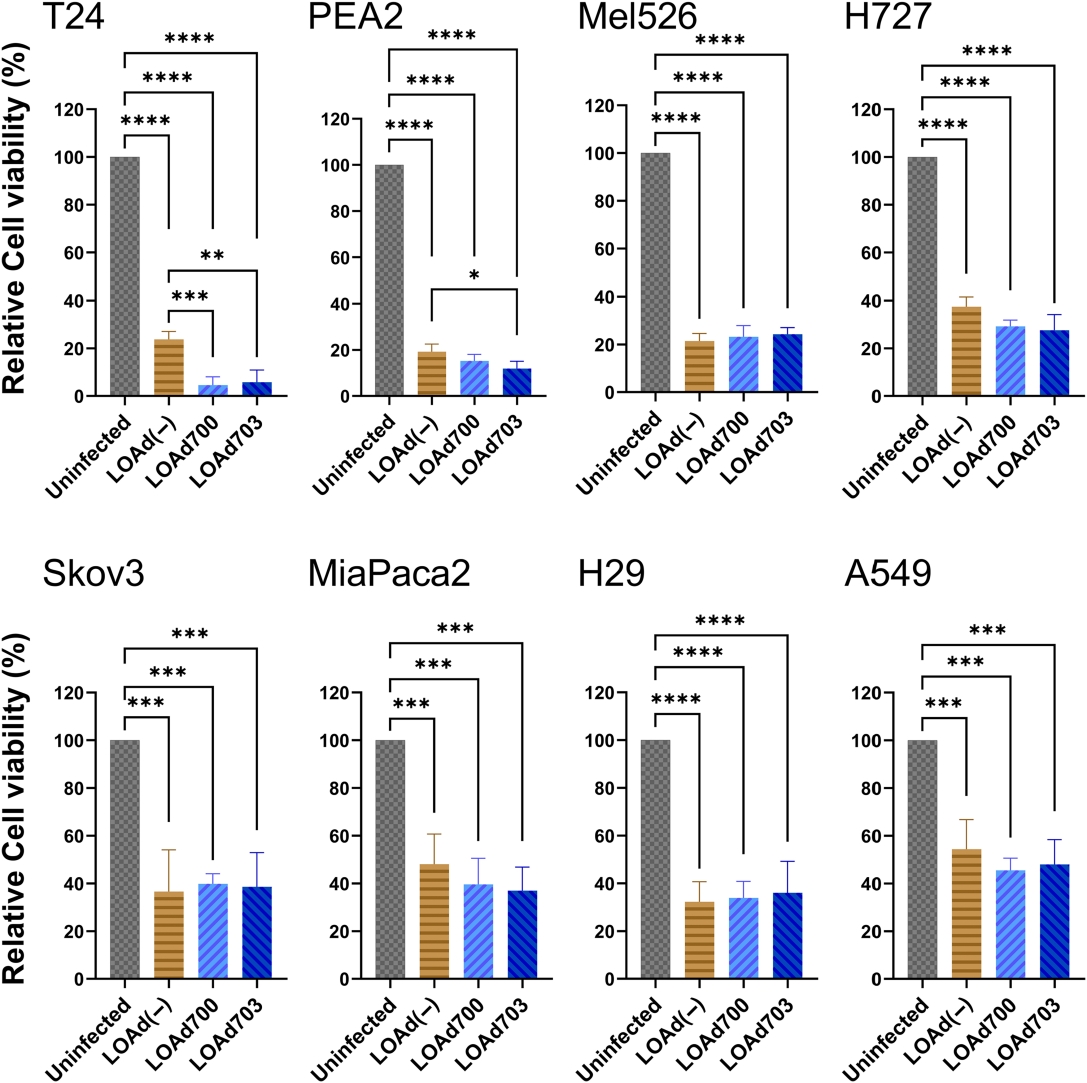
Oncolytic capacity of LOAd viruses. Human cancer cell lines (T24, PEA2, Mel526, H727, Skov3, MiaPaCa2, H29 and A549) were infected with 100 FFU/cell LOAd viruses or left uninfected. Relative cell viability of infected cells was determined at 48 h post infection by MTS assay. Viability is shown as percent of uninfected control (mean ± SD). Data represent three independent experiments. Statistics were calculated with ordinary one‐way ANOVA with Tukey multiple comparisons test (*p ≤ 0.033, **p ≤ 0.0021, ***p ≤ 0.0002, ****p ≤ 0.0001).

**FIGURE 4:**
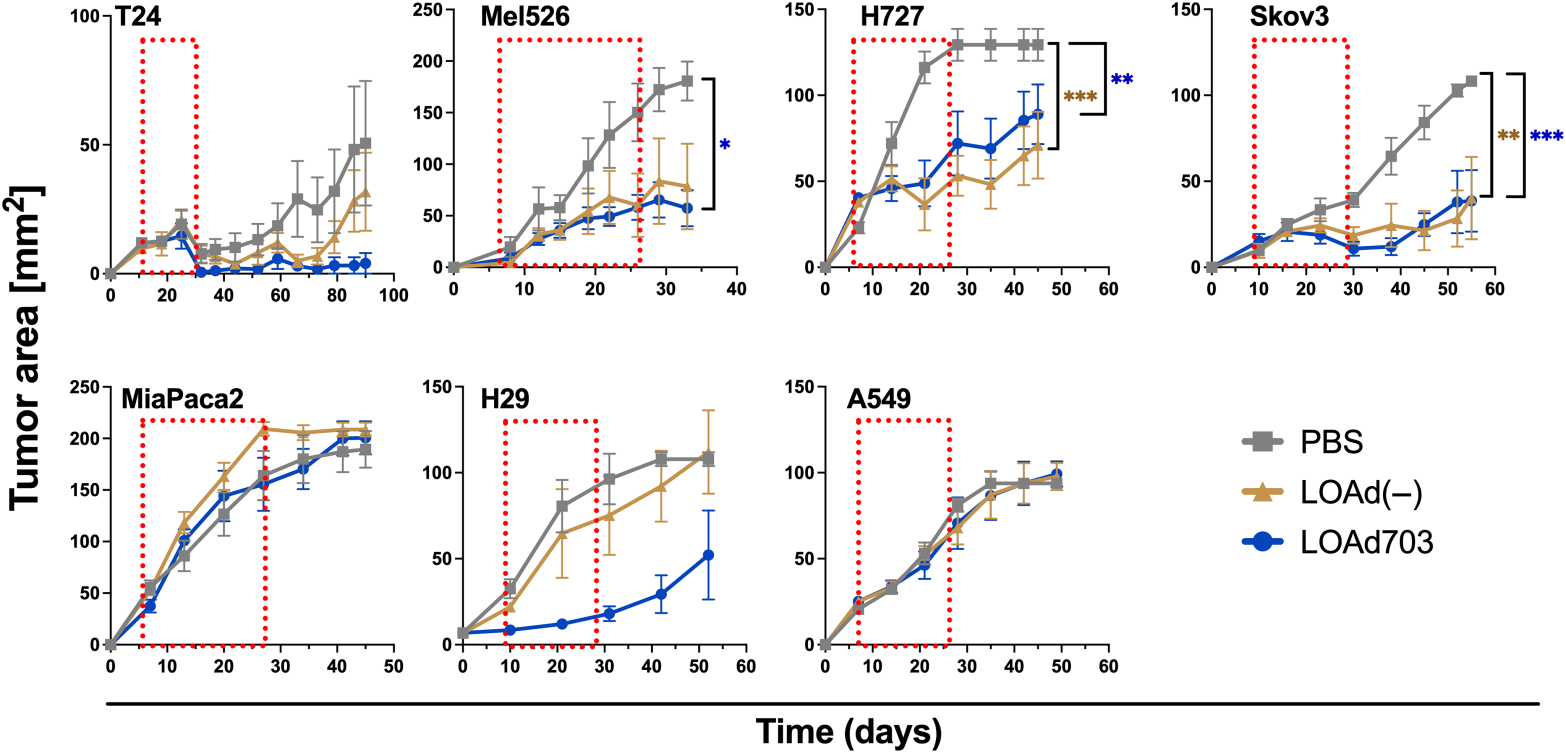
Oncolytic capacity of LOAd viruses in vivo. Human cancer cell lines T24, Mel526, H727, Skov3, MiaPaca2, H29 and A549 were grown in xenograft BalbC Nu/Nu mice (*n* = 5 per group). When tumours were palpable, mice were treated intratumorally with PBS, LOAd(−) or LOAd703 (1 × 10^9^ FFU/mouse) twice weekly for 3 weeks. Graphs display tumour growth curves. Grey lines with rectangle represent PBS‐, brown lines with triangle represent LOAd(−)‐ and blue lines with circle represent LOAd703‐treated groups. Red dashed squares display the time window for LOAd treatment. Statistical differences were calculated with one‐way ANOVA followed by Tukey multiple comparison tests (*p ≤ 0.033, **p ≤ 0.0021, ***p ≤ 0.0002).

LOAd viruses are designed to selectively replicate in cells with a defective retinoblastoma pathway displaying hyperphosphorylation, point mutations or deletions.^10^ To confirm LOAd‐mediated oncolysis, the tumour panel was infected with LOAd703, LOAd700, LOAd(−) or left uninfected. All LOAd viruses reduced the viability of the cells post infection. The cell lines showed similar sensitivity to virus‐mediated oncolysis (Figure 3). Of interest, infecting the T24 CD40^+^ cell line with TMZ‐CD40L‐expressing viruses (LOAd703 and LOAd700) demonstrated a significantly higher oncolytic capacity compared to LOAd(−). In addition, CD40‐positive PEA2, also displayed a significantly lower viability post LOAd703 infection compared to LOAd(−) infection. The oncolytic capability of the viruses was also investigated in vivo in a xenograft model using human tumours in immunodeficient mice as replication only occurs in human cells (Figure 4). Tumours were implanted subcutaneously and when palpable, intratumoral injections with LOAd703, LOAd(−) or vehicle control were initiated. The animals received a total of six injections over 3 weeks. In the T24 model, a trend towards increased killing of LOAd703 treated mice was seen but tumours were small and the variation high in the control group. The PEA2 could not be evaluated in vivo due to poor growth. Hence, we did not gain conclusive data of the in vivo oncolytic capacity and contribution of CD40‐mediated apoptosis. Nevertheless, LOAd703 could control the growth of tumours in 4/6 of the remaining tumour models (Mel526, H727, Skov3 and H29) and LOAd(−) in 3/6 (Mel526, H727 and Skov3) compared to vehicle control. MiaPaca2 and A549 were resistant in vivo to oncolysis even if they were equally sensitive to LOAd viruses in vitro.

### 3.4 TMZ‐CD40L gene transfer mediates killing of CD40 positive tumour cells

**FIGURE 5:**
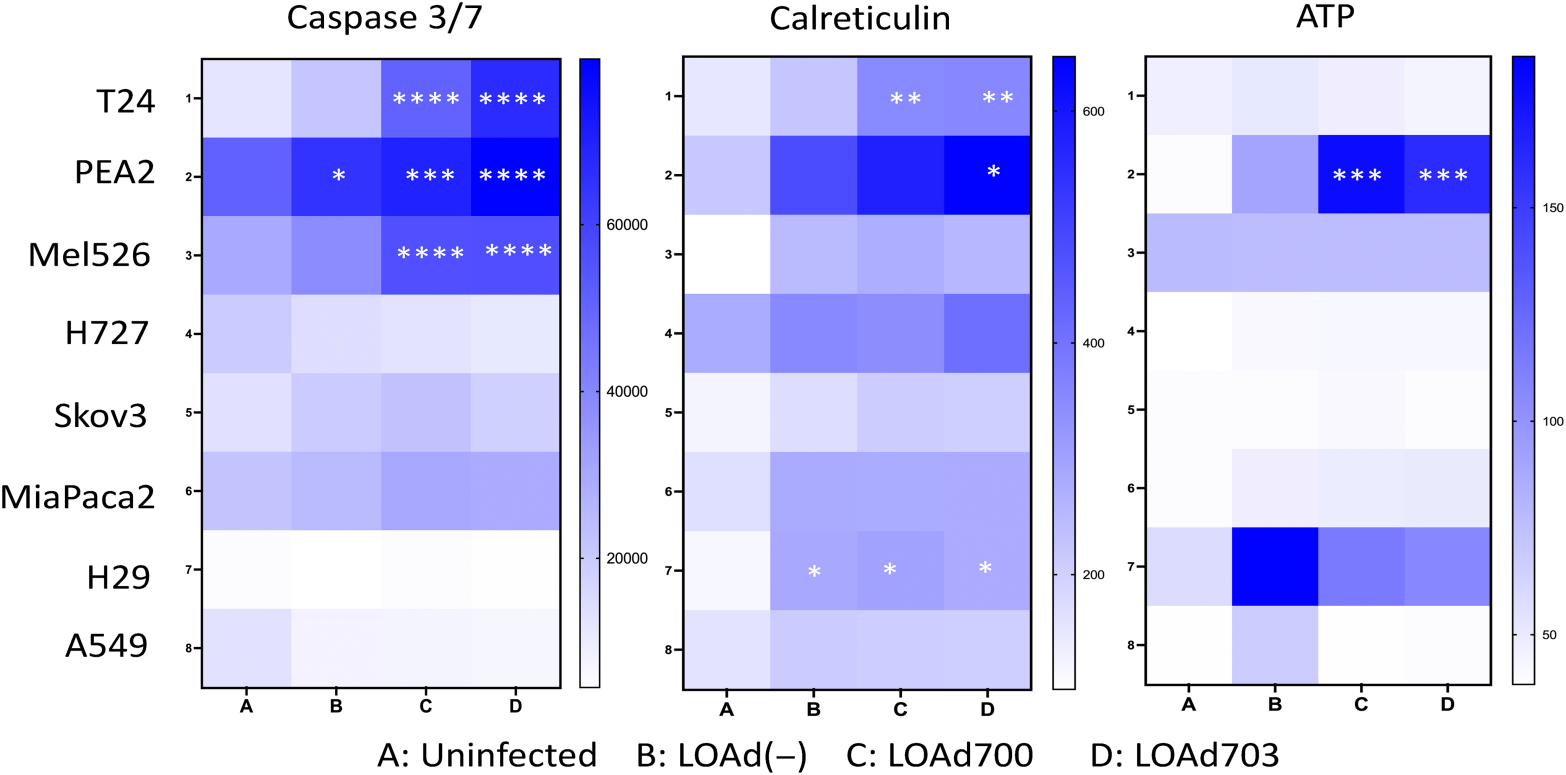
Markers of cell death in LOAd‐infected cells. Cancer cell lines (T24, PEA2, Mel526, H727, Skov3, MiaPaCa2, H29 and A549) were infected with 50 FFU/cell (in caspase activity assay) or 10 FFU/cell (in the other assays) of LOAd viruses or left uninfected. Caspase 3/7 activity was measured at 24 h post infection using the Caspase Glow® 3/7 assay. Cell surface expression of calreticulin was measured 48 h post LOAd infection by flow cytometry. Release of extracellular ATP were measured in supernatant 48 h post LOAd infection by ATP determination assay. Heatmaps demonstrate luminescence (Caspase 3/7, ATP) and mean fluorescence intensity (Calreticulin). Data display three independent replicates for Caspase 3/7 and ATP and two independent replicates for Calreticulin. Statistical comparisons were performed using one‐way ANOVA followed by Tukey's multiple comparison test. The significances displayed represents differences noted to uninfected cells (*p ≤ 0.033, **p ≤ 0.0021, ***p ≤ 0.0002, ****p ≤ 0.0001).

To understand killing mechanisms and immunogenicity of the LOAd‐mediated cell death, the cell panel was tested for apoptosis markers Caspase 3 and 7 as well as calreticulin (‘eat‐me’ signal) and released ATP (‘find‐me’ signal). Five out of eight cell lines increased caspase 3/7 activity post LOAd virus infection suggesting apoptosis is involved in the oncolytic process but not dependent on it (Figure 5A). However, only three of eight had significant changes, among them the two CD40‐positive cell lines. T24 and PEA2 showed a significant increase of active Caspase 3/7 upon infection with LOAd703, and T24 also to LOAd700, compared to LOAd(−). Hence, in these cell lines, cell death may be triggered to a greater extent via apoptosis through CD40L/CD40 signalling. However, also Mel526 showed significant increase of active Caspase 3 and 7 which was pronounced after infection with LOAd700 and LOAd703. Calreticulin is exposed on the cell surface of cells undergoing apoptosis but damaged membrane during oncolysis may also result in positive staining. Post LOAd infection, all cells became positive for calreticulin but only three at significantly increased levels (Figure 5B). The CD40‐positive T24 and PEA2 cells showed a significant increase of calreticulin when expressing TMZ‐CD40L via LOAd700 and/or LOAd703. Calreticulin increase was also significant in H29 post virus infection. ATP release was not altered in most cell lines except PEA2 (Figure 5C). Nevertheless, all markers (Caspase 3/7, calreticulin and ATP) considered, apoptosis is involved in the death mechanisms post LOAd infection especially when LOAd expresses the TMZ‐CD40L.

### 3.5 Immunostimulatory gene therapy targeting CD40 and 4‐1BB upregulates death receptor expression

**FIGURE 6:**
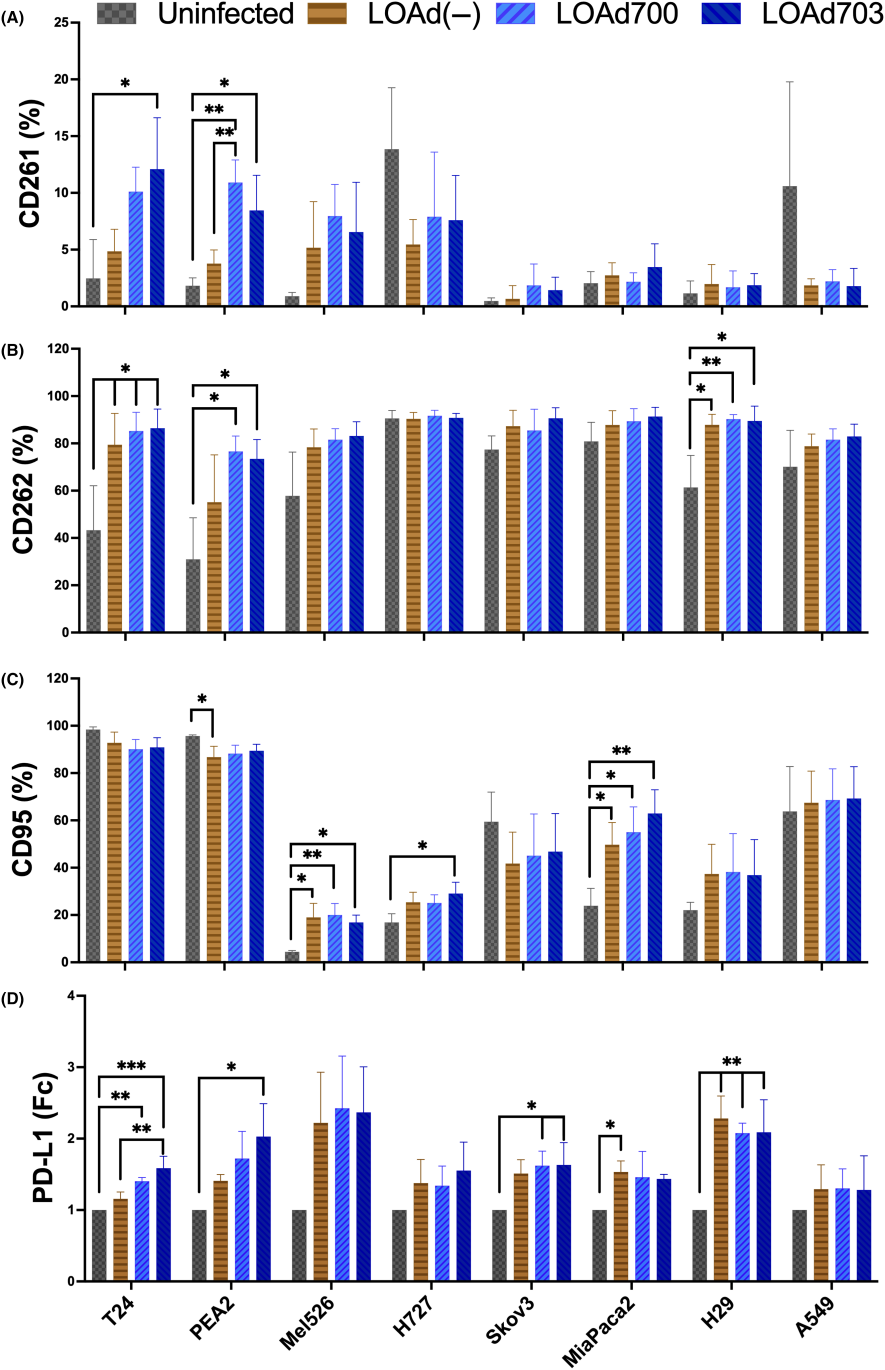
Expression of death receptors and PD‐L1 post LOAd infection. Cancer cell lines (T24, PEA2, Mel526, H727, Skov3, MiaPaCa2, H29 and A549) were infected with 10 FFU/cell of LOAd(−), LOAd700, LOAd703 or left uninfected. After 48 h, cells were analysed for surface expression of markers CD261 (A), CD262 (B), CD95 (C) and PD‐L1 by flow cytometry showing mean fluorescence intensity (MFI) fold change (D). Bar graphs represent % of positive viable cells of three independent replicates. Statistical comparisons were performed using ordinary One‐way ANOVA followed by Tukey multiple comparison test (*p ≤ 0.033, **p ≤ 0.0021).

As LOAd703 is an immunostimulatory gene therapy, it is of interest to understand if infected tumour cells become good targets for immune cell‐mediated killing. Hence, the tumour cells were analysed for expression of ‘kill‐me’ signals such as death receptors TRAIL‐R1 and 2 (CD261 and CD262, respectively) and Fas (CD95). Both TRAIL‐R1 and 2 were upregulated by viral infection but the increase was significant especially upon TMZ‐CD40L expression induced by LOAd700 and/or LOAd703 in the cell lines expressing CD40 (T24 and PEA2) (Figure 6A,B). Hence, targeting the CD40 pathway of CD40^+^ tumour cells may increase the immune‐mediated killing via TRAIL receptors. Of note, TRAIL‐R2 was significantly increased post infection also in H29 cells but the increase of LOAd(−) infection in both H29 and T24 was similar to that of LOAd700 or LOAd703 meaning that the increase was not dependent of CD40 stimulation. Fas was found in almost all cells and highly expressed by both T24 and PEA2, which displayed a slight drop of Fas post infection, but this was only significant in PEA2 post LOAd(−) (Figure 6C). In the other cell lines, Fas expression was less abundant in uninfected cells but it was significantly increased in Mel526, H727 and MiaPaca2 post infection and a trend was noted in H29. As tumour cells may protect themselves from T cell‐mediated killing by expressing PD‐L1, this receptor was also investigated in the cell lines. It was highly expressed by T24 but less abundant in the other cell lines. It was increased post infection with the virus, significant differences post infection were seen in T24, PEA2, Skov3, MiaPaca2 and H29 (Figure 6D).

## 4 DISCUSSION

Oncolytic virotherapy aims to kill tumour cells via oncolysis and to activate a robust anti‐tumour immune response. Tumour immunity is gained by oncolysis‐mediated exposure of tumour antigens to the immune system. However, oncolysis mediated by viruses alone did not translate into broad clinical success thus far but it has been evident that the inflammation following virotherapy can be used to trigger anti‐tumour immunity. To further stimulate this mechanism‐of‐action, oncolytic virotherapy merged with the immunostimulatory gene therapy field by the genetic engineering of the oncolytic viruses to express human immunostimulatory transgenes. The type of virus and type of transgenes will determine the strength and focus of the immune response which may lead to different outcomes in different patients. Knowledge of possible differences in immunogenicity may support the choice of virus and support patient selection.

Mandatory for infection using LOAd viruses is that the tumour cells express the viral entry receptor CD46. All cell lines tested in this study expressed CD46 at high levels meaning that selection of patients based on CD46 expression may not be necessary. LOAd infection led to transgene expression in infected cells and to decreased cell viability due to oncolysis. Two out of the eight cell lines (T24 and PEA2) were positive for CD40, the receptor of TMZ‐CD40L. The CD40 receptor is a member of the TNF receptor superfamily. It is expressed on a variety of cell types including immune cells, epithelial and endothelial cells. Cancers derived from CD40‐positive cells initially express CD40, but it is commonly lost as the tumour progresses.^23^, ^24^ Correspondingly, CD40‐positive tumours correlate with better survival in several cancer indications.^23^, ^25^ Of interest, CD40‐positive cancers were recently shown to correlate to a better response to checkpoint blockade therapy.^26^ However, it is unclear if it was only due to CD40‐expression on the tumour cells or a combination of CD40‐expressing tumour cells with infiltrating CD40‐positive antigen presenting cells. CD40‐CD40L interaction on immune cells has been associated with DC and B cell maturation and T cell priming. Conflicting reports have been published regarding CD40 signalling in different cancer cells, with pro‐tumoral effects reported by some, while most have shown an association to increased apoptosis and tumour growth arrest post CD40 stimuli of cancer cells.^27^, ^28^, ^29^, ^30^, ^31^, ^32^ The anti‐tumoral effects of CD40 have mainly been observed after high levels of CD40 engagement and ligation to the membrane‐bound rather than the soluble form of CD40L.^32^, ^33^, ^34^, ^35^ In line with a previous study,^10^ we could confirm that TMZ‐CD40L was retained on the cell surface of infected cells and that only a small amount was shedded. Interestingly, even though all cell lines were sensitive for LOAd infection, the CD40 positive cell lines, T24 and PEA2, had significantly reduced viability after infection with TMZ‐CD40L‐expressing LOAd viruses, as compared to after infection with LOAd(−), that lacks transgenes. These results suggest that there could be a contribution from apoptosis mediated by CD40 ligation in the mechanism‐of‐action of LOAd703/700‐induced cell death. A previous study demonstrated that ligation of CD40 on tumour cells by membrane‐bound wild type CD40L triggers the intrinsic pathway of apoptosis via activation of initiator Caspase 9 and effector Caspases 3 and 7.^34^ Correspondingly, Caspase 3 and 7 were activated to a higher extent in T24 and PEA2 upon infection with LOAd viruses expressing TMZ‐CD40L. Our data warrant for investigating the CD40 level in the tumours of LOAd703‐treated patients and correlating data to response rate and survival as patients with CD40^+^‐positive cancers may respond better to LOAd703 therapy.

A non‐significant increase of active Caspase 3 and 7 was noted also in other cell lines indicating that it cannot be excluded that apoptosis may be part of the oncolytic process post adenovirus infection. In a previous study conducted by Ma et al., it was shown in the A549 cell line that oncolytic adenoviruses initiate different pathways of cell death including autophagy, inflammasome formation and necroptosis, but not the apoptotic pathway^3^ which was also confirmed in our study. Calreticulin is an intracellular protein located in the cytosol. Upon stress such as induction of apoptosis, calreticulin can be relocated to the cell surface. Hence, calreticulin is considered a marker of immunogenic cell death and is also known to opsonize the cells for DC uptake.^36^, ^37^, ^38^, ^39^ All cell lines tested in this study upregulated calreticulin, but it reached significance in only three of these cell lines including the CD40^+^ T24 and PEA2. However, virus replication and the subsequenct oncolysis may contribute to a disturbed plasma membrane which may expose intracellular calreticulin for staining methods which may explain the background, nonsignificant staining. A similar upregulation of calreticulin post adenovirus infections have been reported.^40^

Another marker associated with immunogenic cell death is extracellular ATP which functions as a chemoattractant of APCs.^41^ Extracellular ATP can also stimulate DCs to upregulate expression of costimulatory receptors, including CD80, CD86 and CD40.^5^ Optimal release of ATP is autophagy activation‐dependent. When autophagy is inhibited, the secretion of ATP is suppressed.^42^ Our analysis of extracellular ATP in supernatants collected from cells treated with LOAd viruses demonstrated that LOAd infections upregulated secretion of ATP in PEA2, MiaPaca2 and H29 but this upregulation was significant only in PEA2. Low upregulation or absence of ATP secretion post LOAd treatment could be due to lack of autophagy in the cell lines. However, the analysed cell supernatants were collected at 48 h post treatment. Since secretion of ATP occurs during early stages of cell death and ATP is unstable when secreted into the extracellular milieu, the collection time point could be too late to capture the peak values.

Death receptor expression enables effector T cells and NK cells to kill the tumour via receptor‐mediated apoptosis. TRAIL‐R1 and 2 were both significantly upregulated post infection with a TMZ‐CD40L‐containing LOAd virus in the CD40‐positive cell lines even if several other lines tended to upregulate these receptors as well. Fas on the other hand was highly expressed by both CD40‐positive cell lines and virus infection did not further augment expression. It was rather downregulated. However, in cell lines with low intrinsic Fas expression such as Mel526, virus infection significantly upregulated Fas.

Finally, we investigated the expression of PD‐L1 on the tumour cell lines pre and post infection due to its role in dampening immune responses. Infection with viruses is known to upregulate the expression of PD‐L1 in infected cells, and we have previously shown that LOAd703 infection of DCs led to upregulation of this marker.^9^, ^43^ All cell lines were positive for PD‐L1 at baseline and infection with LOAd viruses led to upregulation of this marker in most cell lines This could potentially hamper the immunostimulatory effects induced by LOAd703 infection. One way to counteract this effect is to combine LOAd703 with check‐point inhibitors and this concept is currently being investigated in three clinical trials (NCT02705196, NCT04123470 and NCT03555149).

## 5 CONCLUSIONS

**FIGURE 7:**
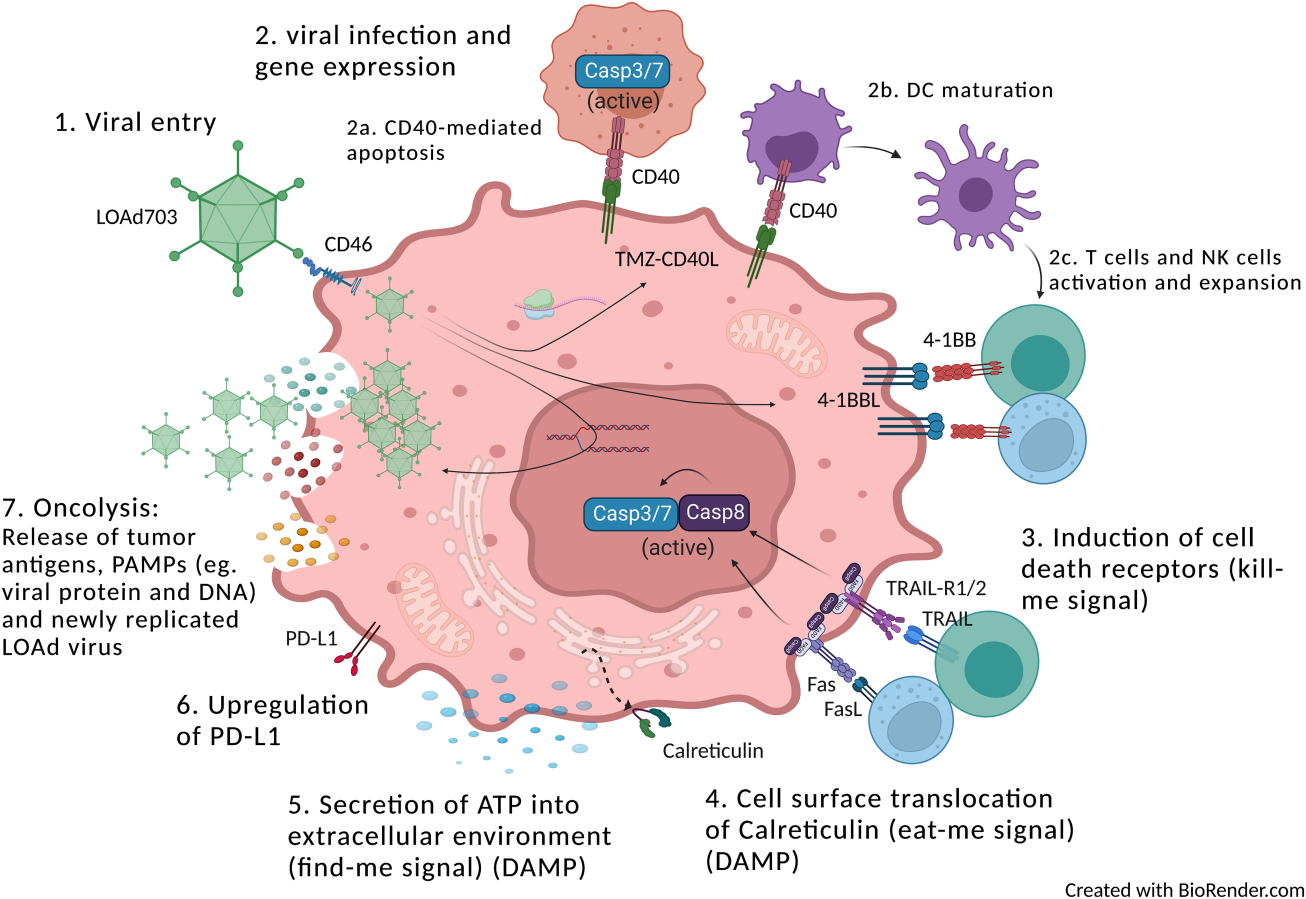
Mechanism‐of‐action of immunogenicity post LOAd703 infection. The proposed mechanism‐of‐action of inducing tumour cell immunogenicity post LOAd703 based on the data presented herein is displayed in this summarized Figure. The Figure also includes the immunostimulatory capacity published previously. The LOAd703 virus enter the cell via the CD46 receptor whereupon the virus will transcribe and translate the human immunostimulatory genes TMZ‐CD40L and 4‐1BBL which will be displayed on the cell surface. CD40 positive tumour cells can be stimulated to undergo CD40‐mediated apoptosis with activation of Caspase 3 and 7 upon TMZ‐CD40L ligation. TMZ‐CD40L and 4‐1BBL can stimulate dendritic cell maturation which in turn leads to T cell activation. 4‐1BB can also stimulate T and NK cell expansion. Virus infection and CD40 stimuli will upregulate death receptors such as TRAIL R1 and 2 as well as Fas which enables T‐ and NK cell‐mediated killing. Virus infection and CD40 stimuli can also induce calreticulin translocation to the cell surface to enhance DC engulfment and thereby antigen presentation of the tumour. Virus infection can in some cell lines induce release of ATP that attracts immune cells to the tumour. LOAd703 infection may also upregulate PDL1 that may reduce T cell ability to tumour cell killing. Finally, the infected tumour cell will die by oncolysis to release new viral particles into the tumour microenvironment.

In conclusion, the results from the current study reveal that LOAd viruses exhibit oncolytic capacity across cancer indications independent of cancer cells' preference of cell death such as apoptosis or autophagy. Furthermore, LOAd infection leads to transgene expression which can increase the intrinsic tumour immunogenicity by upregulating expression of death receptors and immunological cell death markers. These effects were potentiated when CD40L‐armed viruses, infected CD40‐positive cancer cells. Hence, CD40 may be a selection marker for LOAd703 therapy. However, the immune‐mediated killing may be hampered by upregulation of PDL1 and LOAd703 therapy might benefit from simultaneous PDL1 blockade. A proposed LOAd703 mechanism‐of‐action based on current and previous data is shown in Figure 7.

## AUTHOR CONTRIBUTIONS

**Sedigheh Naseri:** Data curation (lead); formal analysis (lead); investigation (lead); methodology (equal); writing – original draft (lead). **Mariela Mejia Cordova:** Data curation (supporting); methodology (supporting); writing – review and editing (supporting). **Jessica Wenthe:** Data curation (supporting); formal analysis (supporting); visualization (supporting); writing – review and editing (supporting). **Tanja Lövgren:** Conceptualization (supporting); methodology (supporting); visualization (supporting); writing – review and editing (supporting). **Emma Eriksson:** Conceptualization (lead); formal analysis (equal); methodology (equal); project administration (equal); supervision (supporting); visualization (supporting); writing – review and editing (equal). **Angelica Loskog:** Conceptualization (equal); funding acquisition (lead); supervision (equal); visualization (supporting); writing – original draft (equal); writing – review and editing (lead). **Gustav J. Ullenhag:** Conceptualization (equal); funding acquisition (equal); supervision (lead); writing – review and editing (equal).

## FUNDING INFORMATION

These studies were supported by grants to Dr Loskog and Dr Ullenhag from the Swedish Cancer Society, grant numbers 200756PjF and 19 0421, respectively, to Dr Loskog from the Childhood Cancer Society, grant number PR2021‐0061 and The Swedish Research Council, grant number 2019/01721. The study was also funded partly by a contract research agreement between Uppsala University and Lokon Pharma AB.

## CONFLICT OF INTEREST STATEMENT

AL is the CEO and board member of Lokon Pharma. EE and JW are employees of Lokon Pharma. The other authors have no conflicts of interest to disclose in relation to this manuscript. There is a patent in regards to the work held by Lokon Pharma (WO2015155174A1).

## Data Availability

The data that support the findings of this study are available from the corresponding author upon reasonable request.
